# Implementation and First Results of the Czech Nationwide Prostate Cancer Screening Pilot Program

**DOI:** 10.1016/j.euros.2026.07.003

**Published:** 2026-07-25

**Authors:** Marcela Koudelková, Kateřina Hejcmanová, Marek Babjuk, Roman Zachoval, Jiří Ferda, Ola Bratt, Renata Chloupková, Ondřej Ngo, Karel Hejduk, Vlastimil Válek, Ladislav Dušek, Ondřej Májek

**Affiliations:** a National Screening Centre, Institute of Health Information and Statistics of the Czech Republic, Prague, The Czech Republic; b Institute of Biostatistics and Analyses, Faculty of Medicine, Masaryk University, Brno, The Czech Republic; c Department of Urology, Second Faculty of Medicine, Charles University, Motol University Hospital, Prague, The Czech Republic; d Department of Urology, Third Faculty of Medicine, Charles University and Faculty Thomayer Hospital, Prague, The Czech Republic; e Department of Imaging Methods Faculty of Medicine in Pilsen, Pilsen, The Czech Republic; f Department of Urology, Institute of Clinical Sciences, Sahlgrenska Academy, University of Gothenburg, Gothenburg, Sweden; g Department of Urology, Sahlgrenska University Hospital, Gothenburg, Sweden; h Department of Radiology and Nuclear Medicine, Masaryk University, Brno, The Czech Republic; i RECETOX, Faculty of Science, Masaryk University, Brno, The Czech Republic

**Keywords:** Cancer screening, Early detection, Population, Prostate cancer, Prostate-specific antigen, Screening

## Abstract

**Background:**

In 2022, the European Union recommended piloting organized prostate cancer screening. Subsequently, the Czech Republic launched a nationwide pilot program fully reimbursed by public health insurance in January 2024 to reduce the high incidence of late-stage prostate cancer and the widespread unorganized prostate-specific antigen (PSA) testing.

**Objective:**

To describe the strategy, methodology, and first results of the Czech nationwide prostate cancer screening pilot program.

**Design, setting, and participants:**

We analyzed data from the first 12 mo of the program targeting men aged 50–69 yr without a previous diagnosis of prostate cancer. General practitioners and urologists offer men an initial PSA test. Those with PSA ≥3.0 µg/l are referred to certified urologists. Based on urology assessment, selected for magnetic resonance imaging (MRI) and, if indicated, targeted biopsy.

**Outcome measurements and statistical analysis:**

Reported key indicators are PSA testing coverage and overall use of prebiopsy MRI.

**Results and limitations:**

Screening was offered to 150 498 men; PSA results were available for 146 109 (97.1%) men, 8.8% of whom had PSA ≥3.0 µg/l. Within 6 mo, 67.2% of the men with PSA ≥3.0 µg/l underwent urological evaluation. From 2023 to 2024, the 2-yr PSA testing rate rose from 47.4% to 53.3%, and prebiopsy MRI use increased from 28.0% to 38.3% (10.3 percentage-point difference, 95% confidence interval 8.3–12.2). Data on diagnostic outcomes are not yet analyzed.

**Conclusions:**

These results demonstrate the feasibility of transforming widespread unorganized PSA testing into an organized screening program with improved adherence to the recommended diagnostic pathway. Further evaluation to assess outcomes and efforts to reduce opportunistic testing will follow.

## Take Home Message

Organized prostate cancer screening is feasible at the national level. Early results show increased prostate-specific antigen testing coverage and better alignment with evidence-based diagnostic practices.

ADVANCING PRACTICE **What does this study add?**

This is the first population-level evaluation of a nationwide organized prostate cancer screening program in Central Europe. The findings demonstrate the feasibility of integrating a structured screening program with prostate-specific antigen testing in routine primary care into a national health system. The program improved adherence to guideline-based diagnostic pathways.

**Clinical Relevance**

This study demonstrates that transitioning from widespread opportunistic PSA testing to an organized screening program is feasible at a national level and can improve adherence to recommended diagnostic pathways, including increased use of prebiopsy MRI. The high participation rates should be interpreted in the context of the Czech invitation strategy, in which screening was actively offered by general practitioners and urologists during routine care rather than through a population-based invitation system. Further evaluation is needed to determine the program's impact on cancer detection, clinical outcomes, and its generalizability to population-based prostate cancer screening. Associate Editor: Roderick C.N. van den Bergh, MD PhD.

**Patient Summary**

This report presents the initial results of a new nationwide, organised prostate cancer screening program in the Czech Republic. The program contributed to increased screening coverage in the target population (50–69 yr) and improved the use of MRI before biopsy, which is now the recommended practice.

## 1 Introduction

Prostate cancer is one of the most common malignancies among men in the European Union (EU), and the incidence is steadily increasing [1]. Late diagnosis is a persistent challenge, with approximately one-third of cases detected as locally advanced (T3–T4, N0, and M0) or metastatic (N1/M1) disease in the Czech Republic [2], when therapeutic options are limited, and the prognosis is poor [2]. In contrast, early detection significantly increases the chance of curative treatment and long-term survival [3], [4]. Approximately 8000 new prostate cancer cases are diagnosed annually, nearly three times more than those diagnosed two decades ago [2]. This rise is attributable not only to the ageing population but also to the widespread use of opportunistic prostate-specific antigen (PSA) testing. International randomized trials have demonstrated that organized PSA-based screening can reduce prostate cancer mortality but leads to overdiagnosis and overtreatment [4], although less so than unorganized testing [4], [5]. Screening may thus lead to clinically unjustified diagnostic interventions and put an unnecessary burden on health care services [6], [7]. In response to current challenges of unorganized testing, the EU in 2022 recommended that member states assess the feasibility and effectiveness of organized screening programs [8], endorsed by the 2022 International Prostaforum declaration that supported the launch of pilot screening programs across Europe [9].

Based on these frameworks, a nationwide prostate cancer screening pilot program was launched in the Czech Republic in January 2024 [10]. The primary objective of the program is to assess the feasibility of organized PSA screening to guide diagnostics and reduce advanced prostate cancer. The program also validates a structured early detection algorithm incorporating PSA-based risk stratification and magnetic resonance imaging (MRI)–guided biopsy within the Czech health care system [11].

We here describe the methods used in the Czech national prostate cancer screening program and its initial outcomes.

## 2 Patients and methods

### 2.1 Target population

The nationwide prostate cancer screening pilot targets men aged 50–69 yr with no prior prostate cancer or clinical suspicion. Men aged >70 yr are not actively enrolled but may be offered the same scheme based on individual health at the physician’s discretion.

### 2.2 Enrollment and diagnostic pathway

**Fig. 1:**
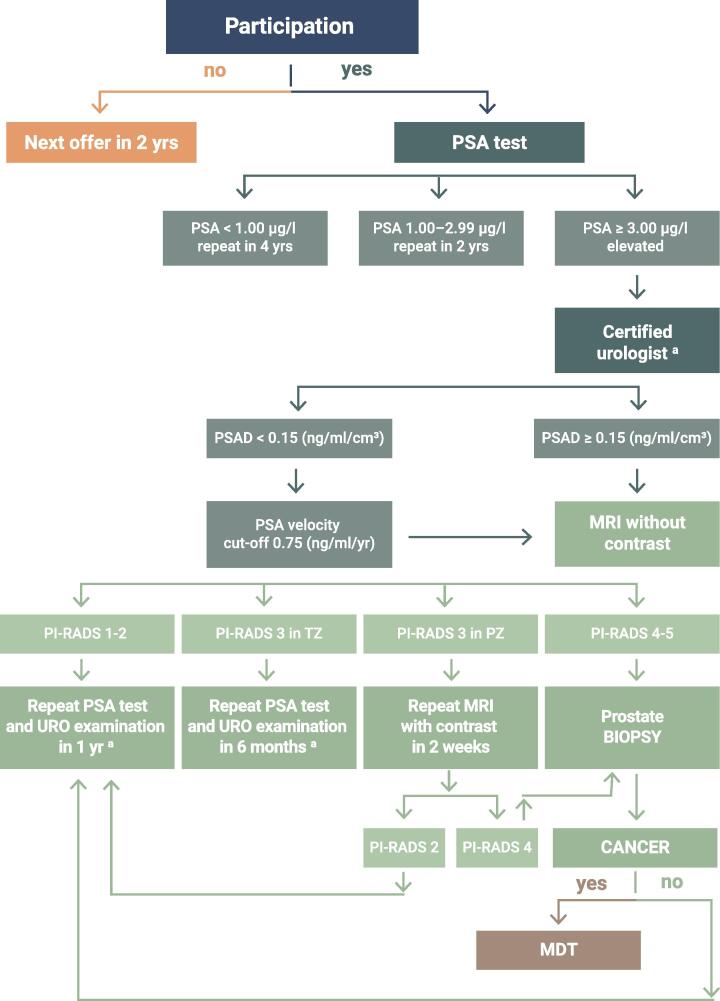
Screening algorithm. MDT = multidisciplinary team; MRI = magnetic resonance imaging; PI-RADS = Prostate Imaging Reporting and Data System; PSA = prostate-specific antigen; PSAD = prostate-specific antigen density; PZ = peripheral zone; TZ = transition zone; URO = urology. ^a^Includes a series of clinical examinations defined by the program algorithm (Supplement).

The screening algorithm is based on international evidence-based recommendations and consensus of relevant professional societies [12], [13], [14], incorporating specifics of the Czech health care system. Details are provided in Figure 1 and the Supplementary Material. General practitioners (GPs) enroll men, typically at routine biennial preventive check-ups. Urologists may enroll patients attending for other urological conditions. After providing information and obtaining oral consent, a PSA test is performed. Management is stratified by PSA: <1.0 µg/l, retest in 4 yr; PSA 1.0–2.99 µg/l, retest in 2 yr; PSA ≥3.0 µg/l, referral to a Czech Urological Society–certified urologist for further assessment (Supplement). If indicated (Fig. 1 and Supplement), men undergo biparametric MRI at a Ministry of Health–certified center. MRI is interpreted using Prostate Imaging Reporting and Data System (PI-RADS): PI-RADS 1–2, return to urologist for repeat PSA assessment in 1 yr; PI-RADS 3, repeat multiparametric MRI with contrast (peripheral zone lesion) or PSA retest in 6 mo (transitional zone lesion); and PI-RADS 4–5, image fusion biopsy at a certified center. Men with prostate cancer are referred to a multidisciplinary team, preferably at a certified onco-urological center.

### 2.3 Data sources and statistical methods

Patient-level data were obtained from the Czech National Health Information System [15], [16], including the National Registry of Reimbursed Health Services (NRRHS) that covers reimbursed care from Czech health insurance companies, and the Czech National Cancer Registry (CNCR). Data for 2024 and 2025 were still preliminary and will be further validated. Demographic data from the Czech Statistical Office were used to determine the target population.

The primary objective was to describe the number of men who were approached to enter the program by GPs and urologists, participation rates, PSA distribution, diagnostic measures, and cancer detection. As national registries do not yet collect exact values, only PSA categories were available: <1 µg/l, 1–2.99 µg/l, or ≥3 µg/l. Results were stratified by 5-yr age groups.

Enrollment and PSA data were assessed for the first year of the program (January through December 2024). For men with PSA ≥3 µg/l, urologist follow-up within 6 mo, MRI within 9 mo, and prostate biopsy within 9 mo were assessed. Prostate cancer registration in the CNCR within 1 yr of PSA ≥3 µg/l was assessed. Prostate cancer detection in the CNCR within 1 yr of PSA ≥3 µg/l was an additional measure, although cautious interpretation is needed because of the preliminary nature of the source data. The primary objective was to describe the number of men who were approached to enter the program by GPs and urologists, participation rates, PSA distribution, diagnostic measures, and prostate cancer detection (International Classification of Diseases, 10th Revision (ICD-10) diagnosis C61, all grades together). Because of data availability, follow-up indicators were only assessed in men with a PSA result in January–June 2024.

The second objective was to analyze how the organized screening program affects opportunistic PSA testing. PSA testing coverage was defined as the number of men aged 50–69 yr with any PSA test in the past 2 yr divided by the target population size. For example, coverage for 2023 includes tests performed in 2022 and 2023. Men who died before the year for which the indicator was evaluated were not included in the coverage. All men who turned 50 yr in 2024 were categorized as aged 50 yr. To assess whether preexisting secular trends could explain the observed differences between the pre- and postimplementation periods, we evaluated the association between calendar year and PSA testing coverage during the preimplementation period (2011–2023) using linear regression. The estimated annual change was then compared with the observed change between 2023 and 2024.

The third objective was to assess adherence to diagnostic guidelines before and after screening program initiation. Men aged 50–69 yr who underwent PSA testing (by GPs or urologists) in the first half of 2023 and 2024 (within or outside the program) were monitored for a subsequent prostate biopsy and whether an MRI preceded or followed the biopsy. Two-sided 95% confidence intervals (CIs) were calculated for proportions and their differences.

## 3 Results

### 3.1 Recruitment and baseline PSA testing

In its first year (2024), 150 498 men aged 50–69 yr were enrolled in the Czech national prostate cancer screening pilot by 3291 GPs (97.1% of the men) and 89 urologists.

PSA results were available for 146 109 men; 1501 declined participation, and 2888 had unknown status. Among men with known outcome, 99.0% participated (Supplementary Fig. 1).

The largest 5-yr age group was 50–54 yr (33.5%). PSA <1.0 µg/l was recorded in 56.5% of all men (*n* = 82 613), in 66.5% of the men aged 50–54 yr, and in 44.4% of the men aged 65–69 yr. PSA ≥3.0 µg/l was recorded in 8.8% (*n* = 12 874) of the men, from 4.0% to 15.7% across 5-yr age groups (Table 1).

**Table 1:** Age distribution of men approached for the screening program and PSA results by 5-yr age groups. Data source: National Registry of Reimbursed Health Services, January–December 2024

Age (yr)	50–54	55–59	60–64	65–69
Screening offer, *n* (row-wise proportion)	50 405 (33.5)	38 103 (25.3)	33 124 (22.0)	28 866 (19.2)
PSA screening results available^a^, *n*	48 988	37 033	32 176	27 912
PSA <1.0 µg/l, *n* (column-wise proportion)	32 599 (66.5)	21 480 (58.0)	16 137 (50.2)	12 397 (44.4)
PSA 1.0–2.9 µg/l, *n* (column-wise proportion)	14 453 (29.5)	12 798 (34.6)	12 234 (38.0)	11 137 (39.9)
PSA ≥3 µg/l, *n* (column-wise proportion)	1936 (4.0)	2755 (7.4)	3805 (11.8)	4378 (15.7)

PSA = prostate-specific antigen.

a Screening results with known PSA values are presented. For <2% of the men, the result is unknown because of incorrect reporting of data to health care payers, and another 1% chose not to be screened (Supplementary Fig. 1).

Of the 6595 men with PSA ≥3.0 µg/l between January and June 2024, 4431 (67.2%) underwent a urological examination within 6 mo. This proportion declined slightly with age, from 69.9% in the youngest 5-yr age group to 64.8% in the oldest. Within 9 mo, 1270 men (19.3%) underwent MRI and 1104 (16.7%) underwent prostate biopsy. Prostate cancer was detected in 700 men (10.6%), increasing with age from 8.4% in the youngest to 11.6% in the oldest group (preliminary data; Supplementary Table 1).

### 3.2 Transition from opportunistic to organized screening: Results on a population level

**Fig. 2:**
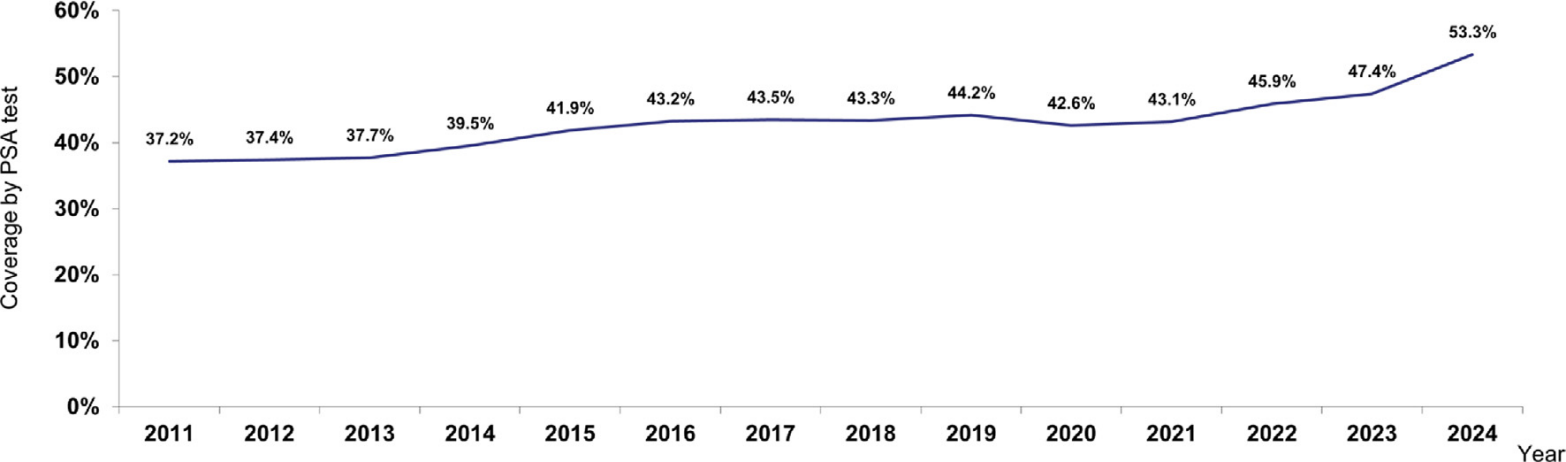
Coverage of the target population by the PSA test in a 2-yr interval in men aged 50–69 yr. Data source: National Registry of Reimbursed Health Services. PSA = prostate-specific antigen.

At the end of 2024, 53.3% of the men aged 50–69 yr without a prostate cancer diagnosis had a PSA test recorded in the preceding 2 yr, a 5.9 percentage-point increase from 2023 (47.4%; Fig. 2), as opposed to a 0.77 percentage-point average increase observed in the preceding years 2011–2023 (*p* < 0.001 for the preimplementation secular trend). At enrollment, 65.6% of program participants had a prior PSA test in the NRRHS (2010–2024); this proportion was 49% in men aged 50–54 yr.

**Fig. 3:**
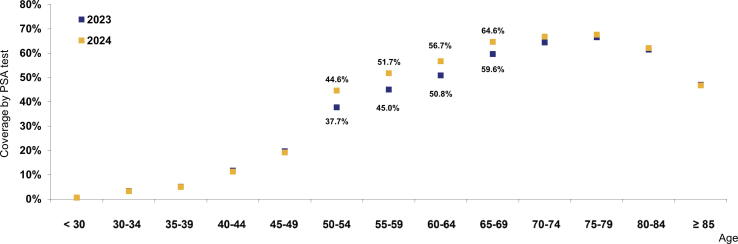
Target population coverage by age for a PSA test in the past 2 yr, in 2023 and 2024, focusing on the 50–69 yr age group. Data source: National Registry of Reimbursed Health Services. PSA = prostate-specific antigen.

PSA testing coverage increased across all age groups following the introduction of the organized screening program in 2024, with the greatest rise observed among men aged 50–54 yr (37.7%–44.6%; Fig. 3).

Adherence to the diagnostic algorithm was assessed over the first 6 mo of the program and compared with the preimplementation period. Prebiopsy MRI increased from 28.0% to 38.3% (+10.3 percentage points; 95% CI 8.3–12.2), whereas postbiopsy MRI decreased by 0.5 percentage points (Table 2).

**Table 2:** Comparison of the proportion of men with MRI before and after prostate biopsy, the first half of 2023 versus the first half of 2024. Data source: National Registry of Reimbursed Health Services

	January–June 2023	January–June 2024
**Men with a PSA result, *n***	227 640	274 887
PSA sampled in primary care, *n*	119 107	164 606
PSA sampled in urology, *n*	108 533	110 281
**Men with a prostate biopsy**^a^, ***n* (%)**	4309 (1.9)	4670 (1.7)
PSA sampled in primary care, *n* (%)	1113 (0.9)	1504 (0.9)
PSA sampled in urology, *n* (%)	3196 (2.9)	3166 (2.9)
**Men with MRI before**^b^**prostate biopsy, *n* (%)**	1208 (28.0)	1789 (38.3)
**Difference between periods, *n* (percentage points; 95% CI)**	10.3 (8.3–12.2)	
PSA sampled in primary care, *n* (%)	217 (19.5)	464 (30.9)
PSA sampled in urology, *n* (%)	991 (31.0)	1325 (41.9)
**Men with MRI after**^c^**prostate biopsy, *n* (%)**	360 (8.4)	371 (7.9)
**Difference between periods, *n* (percentage points; 95% CI)**	0.5 (–1.5–0.7)	
PSA sampled in primary care, *n* (%)	147 (13.2)	177 (11.8)
PSA sampled in urology, *n* (%)	213 (6.7)	194 (6.1)

CI = confidence interval; MRI = magnetic resonance imaging; PSA = prostate-specific antigen.

a Biopsy specimen taken within 180 d after the PSA test.

b MRI done between PSA test and prostate biopsy.

c MRI done within 90 d after prostate biopsy.

## 4 Discussion

This report presents the first results of an organized nationwide prostate cancer screening pilot program in the Czech Republic, targeting men aged 50–69 yr.

### 4.1 Uptake of prostate cancer screening

Overall, 99.0% of the invited men decided to participate. This high uptake is likely attributable to recruitment being conducted directly by GPs and urologists, when men physically attend health care and can undergo PSA testing with minimal extra effort. This personalized approach contrasts with many population-based screening programs and trials that rely on centrally mailed invitation letters. It has been shown that men tend to have a higher level of trust in medical recommendations than in invitation letters [17].

Examples of population-based screening models that rely on mailed invitation letters are the Swedish Gothenburg-1 trial, where participation in the first rounds reached 59% among men aged 50–69 yr [17]; the Swedish regional organized prostate cancer testing (OPT) programs with 35% participation among men aged 50 yr [18]; and the Finnish ProScreen trial with 51% participation among men aged 50–63 yr [19].

The Czech approach builds on recommended regular preventive checkups in primary care that enable GPs to invite men for prostate screening during fully reimbursed visits, to which PSA testing can be added with minimal extra effort. Urologist involvement was limited because their screening billing codes, unlike those for GPs, were not automatically included in insurer contracts; each practice had to apply individually and submit proof of certification, delaying patient enrollment.

Given the incomplete coverage and the biennial cycle of the preventive checkups, the screening program’s first year does not capture the full target population of 1.4 million men. Much of the >50% PSA testing coverage reflects opportunistic testing that cannot yet be fully disentangled from program-driven uptake based on registry data.

### 4.2 PSA results

The observed proportion of 8.8% of men aged 50–69 yr with PSA ≥3 µg/l was in the expected range and so was the increase of this proportion with age [20]. In comparison, 6.7% of men aged 50–60 yr in the Gothenburg-2 trial [21], 12% of men aged 50–74 yr in the STHLM3-MRI trial [14], and 11% of men aged 50–74 yr in the Lithuanian population-based program had a PSA of ≥3 µg/l [22].

### 4.3 Urology assessment

The proportion of men with PSA ≥3 µg/l who underwent the recommended subsequent urological assessment was lower in the first 6 mo of the Czech program (67%) than that in the four Swedish regions with a similar diagnostic pathway for OPT (98–100%) [23]. The Czech proportion may be underestimated, as only 6 mo were analyzed, and waiting times may sometimes be longer. One barrier to urological assessment is the requirement for a certified urologist (lists available on an official website [11]), who may be located some distance away from the participant’s residence. Another factor is that the Czech program has no dedicated coordinators who actively communicate with participants and facilitate appointments, in contrast to Swedish OPT [23]. Consequently, continued participation in the Czech program largely depends on individual motivation. To address this, a centralized national booking system is being developed that will enable direct scheduling for further evaluation and improved continuity of follow-up.

### 4.4 Prostate MRI

The use of prebiopsy MRI and targeted biopsy to reduce unnecessary systematic biopsy and overdiagnosis is crucial in modern prostate cancer screening [24]. After the program initiation, the overall proportion of men undergoing MRI before biopsy increased from 28% in the first half of 2023 to 38% in 2024, indicating improved alignment of diagnostic pathways. This trend temporally correlates with program launch and was accompanied by a concurrent decrease in postbiopsy MRI use; however, the contribution of general clinical trends cannot be excluded. To further improve adherence, educational initiatives targeting urologists and radiologists on program-specific pathways are underway.

MRI capacity is a known bottleneck when implementing screening [25]. In the Czech program, an initial urology assessment selects men for MRI through risk stratification, avoiding direct PSA-to-MRI pathways and protecting MRI capacity, in line with recommendations of the Prostate Cancer Awareness and Initiative for Screening in the European Union (PRAISE-U) project [3], [13]. The impact of this pathway on MRI use and diagnostic outcomes will be analyzed once sufficient longitudinal data are available. Standardized protocols are essential for ensuring high-quality MRI scanning and reading, and subsequent targeted biopsy sampling [25]. The Czech program applies mandatory MRI protocols published by the Ministry of Health (Bulletin 15/2023).

### 4.5 Change of coverage and practice

The observed increase in 2-yr PSA testing coverage from 47% in 2023 to 53% in 2024 (compared to a 0.77 percentage-point average increase observed in preceding years 2011–2023) among men aged 50–69 yr is likely an effect of the organized screening program. Although opportunistic PSA testing persists, the systematic screening pathway has enhanced awareness and accessibility, particularly through GP engagement. The nearly 6 percentage-point increase within a single year suggests successful early program adoption.

In Lithuania, a primary care–based prostate cancer early detection program was initiated in 2006 [22]. Sweden started the implementation of regional, population-based organized testing within public health care in 2020 [23]. This Swedish approach substantially increased the proportion of men aged 50 yr undergoing PSA testing, from 14% to 35% [26]. Notably, unlike the Czech and Lithuanian programs, the Swedish OPT relies on centralized invitations mailed to entire birth cohorts, without any GP involvement.

The planning of the Czech prostate cancer screening program was facilitated by networking with the EU-funded PRAISE-U project [6], coordinating with similarly structured pilot projects in Poland, Ireland, Spain, Lithuania, and Estonia. The Italian Society of Urology has also proposed a structured PSA screening model with defined roles for GPs and specialists [27]. Collaborative development of algorithms and shared performance standards are essential for effective prostate cancer screening in Europe.

### 4.6 Strengths and limitations

The strengths of this report are the program’s nationwide organized design, full public health insurance coverage, and integration into routine care with enrollment at GPs’ preventive checkups, targeting clearly defined age groups for comparability and evaluation. The limitations include preliminary cancer detection data; lack of a dedicated national clinical register (currently under development); low urology follow-up, with only 67% of men with elevated PSA seen by a urologist within 6 mo; and imperfect distinction between organized and opportunistic testing because of code misreporting.

## 5 Conclusions

In 2024, a nationwide prostate cancer screening program was successfully launched in the Czech Republic. Early results show increased PSA testing coverage among men aged 50–69 yr, particularly in the 50–54 yr age group, and greater prebiopsy MRI use consistent with the diagnostic algorithm. These findings indicate that organized screening promotes higher participation and more risk-adapted diagnostic practice. Recruitment through routine preventive checkups, fully covered by public insurance, has proven to be feasible and establishes the foundation for full-scale program implementation. Further evaluation will elaborate on participant and provider compliance with the recommended screening pathway.

  ***Author contributions:*** Ondřej Májek had full access to all the data in the study and takes responsibility for the integrity of the data and the accuracy of the data analysis.

  *Study concept and design*: Koudelková, Májek, Hejcmanová.

*Acquisition of data*: Hejcmanová.

*Analysis and interpretation of data*: Hejcmanová, Májek.

*Drafting of the manuscript*: Koudelková.

*Critical revision of the manuscript for important intellectual content*: Májek, Bratt, Dušek.

*Statistical analysis*: Hejcmanová, Ngo, Chloupková, Májek.

*Obtaining funding*: Májek, Dušek, Hejduk.

*Administrative, technical, or material support*: Hejduk.

*Supervision*: Babjuk, Zachoval, Ferda, Válek, Dušek, Bratt.

*Other* (specify): None.

  ***Financial disclosures:*** Ondřej Májek certifies that all conflicts of interest, including specific financial interests and relationships and affiliations relevant to the subject matter or materials discussed in the manuscript (eg, employment/affiliation, grants or funding, consultancies, honoraria, stock ownership or options, expert testimony, royalties, or patents filed, received, or pending), are the following: None.

  ***Funding/Support and role of the sponsor:*** The study was supported by Programme Johannes Amos Comenius (JAC)–project SALVAGE (CZ.02.01.01/00/22_008/0004644), financed by the Ministry of Education, Youth and Sports (MEYS)—cofunded by the European Union.

The preparatory work for the nationwide pilot program was supported by the project “Complex information background for improving the quality of cancer screening programmes within the National Screening Centre” (CZ.31.8.0/0.0/0.0/23_075/0008430) funded by the European Union from the Recovery and Resilience Facility through the National Recovery Plan of the Czech Republic. This work was supported by the European Union’s Horizon 2020 research and innovation program under grant agreement 857560 (CETOCOEN Excellence). This publication reflects only the authors’ view, and the European Commission is not responsible for any use that may be made of the information it contains.

  ***Acknowledgments:*** The authors would like to acknowledge close collaboration with the PRAISE-U project, which received funding from the EU4Health programme under grant agreement (101101217), cofunded by the European Union. Notably, we are grateful to Hendrik Van Poppel, Sarah Collen, and Monique Roobol-Bouts for the support of the Prague Prostaforum Declaration and preparatory works of the Czech program. They also sincerely thank Eva Nevrtalová for her valuable consultations during the development of this manuscript and Markéta Vranová for her close collaboration in the joint coordination and implementation of the national program in the Czech Republic. The authors thank the RECETOX Research Infrastructure (LM2023069) financed by the Ministry of Education, Youth and Sports, for supportive background.

  ***Data access and responsibility:*** Hejcmanová, K. (2026). Czech nationwide prostate cancer screening pilot programme (DATA) [Data set]. Zenodo. https://doi.org/10.5281/zenodo.18151231.

Preprint of the manuscript is available on https://doi.org/10.64898/2026.01.05.26343424.

## Appendix A Supplementary data

Supplementary Data 1 Additional details of the program's algorithm, the procedure codes used in the prostate cancer screening program, and supplementary analyses supporting the implementation and evaluation of the Czech nationwide pilot prostate cancer screening program are provided in the Supplementary Data.
