# Supplementary material for: Implementation and First Results of the Czech Nationwide Prostate Cancer Screening Pilot Program
Source: Eur Urol Open Sci. 2026 Jul 25;91:41–8. doi: 10.1016/j.euros.2026.07.003 (PMC13445362; doi:10.1016/j.euros.2026.07.003)
Supplement: Supplementary file 1 [file mmc1.docx]

**Supplementary information**

**The National Registry of Reimbursed Health Services (NRRHS) — procedure codes used in the prostate cancer screening programme**

The NRRHS reflects the structure of the Czech national health insurance system, in which coverage is legally mandated for all persons with permanent residence in the Czech Republic, with claims data submitted by all public health insurance companies. This ensures that PSA testing activity — both within the organised programme and opportunistic — is captured comprehensively. A dedicated set of procedure codes was established for the programme in the Ministry of Health Bulletin No. 11/2023, effective from 1 January 2024. The coding logic operates at two levels corresponding to the two tiers of clinical care.

***Primary care level (GPs and, from January 2025, urologists in a primary enrolment role)***

All codes at this level were automatically added to existing GP contracts with all health insurance companies from 1 January 2024, requiring no individual application for the change of the contract. Each enrolment contact is billed as a mandatory pair: a fixed management code combined with exactly one signal code reflecting the outcome — whether PSA was below 1.0, between 1.0–2.99, or ≥3.0 µg/l, or whether the man refused participation. This paired structure ensures that every screened man generates exactly two claims, allowing unambiguous identification of enrolment, PSA result category, and programme dropout in the NRRHS.

***Urology level — requires individual application and certification***

Unlike GP codes, urology-specific screening codes were not automatically contracted. Each urology practice was required to apply individually to each health insurance company and obtain certification from the Czech Urological Society. Two participation tiers were defined: Tier 1 for assessment of men with PSA ≥3 µg/l, and Tier 2 for biopsy centres requiring additional Ministry of Health accreditation. This two-step administrative process — individual contract amendment plus certification — explains why urologist-initiated enrolment remained limited during 2024.

Automatic contracting of urologist-led enrolment codes took effect only from 1 January 2025.

**Detailed description of the programme’s algorithm**

*Enrolment and PSA testing*

Enrolment into the programme is facilitated through GPs (routine preventive health check-ups, which are recommended every two years for all individuals) and through urologists, who invite men already under follow-up for other urological conditions. In both settings, physicians provide education about importance of PSA testing, prostate cancer prevention and inform them about next steps of the programme. In accordance with Czech legislation (Act No. 372/2011), informed consent is needed but not necessarily in written form.

Information on participation is reported by physicians to health insurance companies and subsequently included in the National Registry of Reimbursed Health Services (NRRHS).

If the informed consent is given, a blood sample is collected to determine the serum PSA value.

Follow-up algorithm is based on PSA levels:

**PSA < 1.0 µg/l**:	Repeat PSA screening in 4 years.

**PSA 1.0–2.99 µg/l**: Repeat PSA screening in 2 years.

**PSA ≥ 3.0 µg/l**:	Considered a positive screening result – the man is referred for specialised urological evaluation by a certified urologist.

*Urology assessment*

Only urologist specifically certified by the Czech Urological Society assess screening programme participants. In men with PSA ≥ 3.0 µg/l, a certified urologist carries out examinations according to the programme algorithm:

- repeat PSA test,
- digital rectal examination,
- ultrasonographic examination of the kidneys, urinary bladder,
- transrectal ultrasound (TRUS) for prostate volume measurement,
- calculation of PSA density (PSAD),
- estimation of PSA velocity (PSAV) if more than one PSA test is available.

Based on the findings (PSAD ≥ 0.15 or PSAV cut-off 0.75 ng/ml/year), the urologist may refer for magnetic resonance imaging (MRI), either biparametric (bpMRI, contrast-free) or multiparametric (mpMRI, with intravenous contrast), at an MRI unit certified by the Ministry of Health

In the case of PSA >1 µg/l and a suspicious digital rectal examination, the urologist can initiate either an MRI or a prostate biopsy.

*MRI Evaluation*

**PI-RADS 1 and 2 (negative)**: Further management follows the approved algorithm. The man is returned to certified urologist for, urological examination with a repeat MRI scheduled in one year.

**PI-RADS 3 (equivocal)** in the peripheral zone (PZ): Repeat MRI with intravenous gadolinium-based contrast enhancement (mpMRI) within two weeks. If then PI-**RADS 2 findings**, repeat PSA testing and urological examination after one year. If **PI-RADS 4**, targeted biopsy with MRI/ultrasound image fusion.

**PI-RADS 3** (equivocal) in the transitional zone (TZ): Repeat PSA test and urological examinations (TRUS, PSAD, PSAV) after six months; additional mpMRI or prostate biopsy (high suspicion) if considered indicated.

**PI-RADS 4 and 5 (positive)**: MRI/ultrasound fusion biopsy at a certified biopsy centre. Certification is granted by the Ministry of Health based on established criteria.

Men diagnosed with prostate cancer are referred to a multidisciplinary team for further management. If the biopsy is negative, follow-up MRI is scheduled after one year.

*MRI quality assurance*

MRI quality assurance is a core component of the Czech programme design. MRI examinations may only be performed at accredited radiology centres that have been awarded a certificate by the Ministry of Health, confirming compliance with mandatory technical and procedural standards. These standards — including the protocol for biparametric MRI (bpMRI) of the prostate — were published by the Ministry of Health in Bulletin No. 15/2023 and cover imaging acquisition parameters, reporting requirements, and PI-RADS scoring. Accreditation of radiology centres is an ongoing process administered by the Ministry of Health through successive application rounds; by the end of 2024, four rounds had been completed, with a fifth round opened in early 2026, reflecting the programme's continued expansion of accredited MRI capacity. Accreditation criteria have been iteratively refined based on recommendations from the expert commission overseeing the programme, ensuring that quality standards evolve in line with clinical evidence and operational experience.

**Supplementary Table 1:** Proportion of men with PSA ≥ 3 µg/l and a follow-up examination by a urologist within 6 months, MRI examination within 9 months, prostate biopsy within 9 months, and prostate cancer within 1 year, by 5-year age group. Data source: National Registry of Reimbursed Health Services, Czech National Cancer Registry

| **Age (years)** | **50**–**54** | **55**–**59** | **60**–**64** | **65**–**69** | **Total** |
| --- | --- | --- | --- | --- | --- |
| Men with PSA ≥ 3 µg/l, N | 1,000 | 1,412 | 1,945 | 2,238 | 6,595 |
| Men with PSA ≥ 3 µg/l examined by urologist, N (%) | 699 (69.9) | 964 (68.3) | 1,317 (67.7) | 1,451 (64.8) | 4,431 (67.2) |
| Men with PSA ≥ 3 µg/l with MRI examination, N (%) | 212 (21.2) | 304 (21.5) | 379 (19.5) | 375 (16.8) | 1,270 (19.3) |
| Men with PSA ≥ 3 µg/l with prostate biopsy, N (%) | 147 (14.7) | 249 (17.6) | 337 (17.3) | 371 (16.6) | 1,104 (16.7) |
| *Men with PSA ≥ 3 µg/l with prostate cancer, N (%) | 84 (8.4) | 132 (9.3) | 225 (11.6) | 259 (11.6) | 700 (10.6) |

*Preliminary data, not yet validated; subject to change

**Supplementary Figure 1**: Participation rate, PSA results in the first 12 months, and urological follow-up within 6 months. Data source: National Registry of Reimbursed Health Services, January–June 2024 only. Men enrolled in July–December 2024 (n=6,279) were excluded from the follow-up analysis due to incomplete registry data at the time of analysis; absence of follow-up in this subgroup should not be interpreted as confirmed non-attendance.
